# Negative Pressure Wound Therapy on Surgical Site Infections in Women Undergoing Elective Caesarean Sections: A Pilot RCT

**DOI:** 10.3390/healthcare2040417

**Published:** 2014-09-30

**Authors:** Wendy Chaboyer, Vinah Anderson, Joan Webster, Anne Sneddon, Lukman Thalib, Brigid M. Gillespie

**Affiliations:** 1 NHMRC Centre of Research Excellence in Nursing (NCREN), Centre for Health Practice Innovation, Griffith Health Institute, Griffith University, Gold Coast Campus, QLD 4222, Australia; E-Mails: v.anderson@griffith.edu.au (V.A.); joan.webster@health.qld.gov.au (J.W.); b.gillespie@griffith.edu.au (B.M.G.); 2 Centre for Clinical Nursing, Royal Brisbane and Women’s Hospital, Butterfield Street, Herston, QLD 4029, Australia; 3 Women’s and Newborn Health, Gold Coast University Hospital, Southport, QLD 4215, Australia; E-Mail: anne.sneddon@health.qld.gov.au; 4 Department of Community Medicine (Biostatistics), Faculty of Medicine, Kuwait University, PO Box 24923, Safat 13110, Kuwait; E-Mail: lthalib@hsc.edu.kw

**Keywords:** obesity, surgical site infection, NPWT, caesarean section

## Abstract

Obese women undergoing caesarean section (CS) are at increased risk of surgical site infection (SSI). Negative Pressure Wound Therapy (NPWT) is growing in use as a prophylactic approach to prevent wound complications such as SSI, yet there is little evidence of its benefits. This pilot randomized controlled trial (RCT) assessed the effect of NPWT on SSI and other wound complications in obese women undergoing elective caesarean sections (CS) and also the feasibility of conducting a definitive trial. Ninety-two obese women undergoing elective CS were randomized in theatre via a central web based system using a parallel 1:1 process to two groups *i.e.*, 46 women received the intervention (NPWT PICO™ dressing) and 46 women received standard care (Comfeel Plus^®^ dressing). All women received the intended dressing following wound closure. The relative risk of SSI in the intervention group was 0.81 (95% CI 0.38–1.68); for the number of complications excluding SSI it was 0.98 (95% CI 0.34–2.79). A sample size of 784 (392 per group) would be required to find a statistically significant difference in SSI between the two groups with 90% power. These results demonstrate that a larger definitive trial is feasible and that careful planning and site selection is critical to the success of the overall study.

## 1. Introduction

Between 187 and 281 million surgical procedures are performed around the world each year, or one for every 25 people [1]. In Australia in 2008/9, 1.8 million elective surgeries were performed with one elective surgery for every 12.4 people [2]. Surgical site infections (SSIs) are defined by the Centers for Disease Control and Prevention (CDC) as infections occurring up to 30 days after surgery that affect the incision, deep tissue at the operation site or involve the organs or body space [3]. SSIs have many negative effects including pain, increasing the risk of morbidity and mortality, prolonging hospitalisation and increasing costs [4,5]. Of concern is that SSIs occur in up to 30% of all surgical procedures, and are the third most commonly reported hospital acquired infection [3]. Obesity is an independent predictor of SSI [4,6], thus it has significant safety and cost implications.

Obesity, defined as a body mass index (BMI) ≥30, is a growing global public health problem in developed nations. In 2007–2008, 28%–43% of 18–44 year old Australian women of childbearing age were obese [7]. Obese women are more likely to have a caesarean section (CS). One meta-analysis of 16 studies identified the odds ratio for overweight or obese women (BMI ≥ 25) having a CS as 2.0 (95% CI 1.9–2.2) compared to non-overweight women [8], similar to results of an Australian analysis of 11,252 women giving birth [9]. Post-operative infection is a potential complication of all surgeries including CS, but overweight and obese women are at particular risk [10]. A meta-analysis of 6 studies showed the odds ratio for overweight or obese CS women having an infection was 3.3 (95% CI 2.7–4.1) compared to non-overweight women [8], consistent with individual studies [11]. Given that SSI extends hospital length of stay by up to 6 days in women undergoing obstetric and gynaecologic surgery, increasing hospital costs by US$14,000 for each SSI [12], it has significant implications for women and the health system.

Negative Pressure Wound Therapy (NPWT), also known as vacuum assisted closure, has been used to aid healing since the late 1990s [13,14]. It is based on a closed sealed system that applies negative pressure to the wound surface. The wound is covered or packed with an open-cell foam or gauze dressing and sealed with an occlusive drape. Intermittent or continuous suction is maintained by connecting suction tubes from the wound dressing to a vacuum pump and liquid waste collector. Standard negative pressure rates are 50–125 mm Hg [15]. Despite limited evidence of its effectiveness [16], Tipton and colleagues report “vacuum therapy can be included as an option for management of abdominal wounds, but evidence from randomized controlled trials in obese women undergoing cesarean is not available” [17,18]. Others note NPWT is increasingly being used in closed incisions to prevent SSI [19] and dehiscence. Additionally, one retrospective cohort study of 48 women receiving standard dressings compared to 21 women receiving NPWT found fewer wound complications in the NPWT group, but this difference was not statistically significant [18]. Limitations of Mark *et al*.’s study [18] such as the small sample size, use of historical controls, lack of control over the dressings used in the control group and reliance on coded medical record data suggests the findings should be interpreted very cautiously. Finally, a recent Cochrane Review of NPWT notes limited evidence for its effectiveness and recommends high quality trials to be undertaken [16]. Thus, this limited evidence base became the impetus to undertake a pilot trial in preparation for a larger, definitive trial of NPWT in obese women undergoing elective CS.

## 2. Aim

The aim of this pilot randomized controlled trial (RCT) was to assess the feasibility of conducting a larger trial in terms of measurement of potential outcomes, recruitment, intervention fidelity and retention. The hypothesis tested was “In obese women undergoing elective CS, those who receive a NPWT dressing will have significantly better outcomes than those receiving the standard dressing”. Data from this pilot study will assist researchers to determine sample size requirements and potential primary and secondary outcomes to be used in a larger, definitive trial.

## 3. Methods

A parallel group pilot RCT was undertaken (Australian and New Zealand Trial Registation number ACTRN12612000171819). Ethics approval was granted by the hospital and university office of human research ethics committees. An interim analysis of the first 48 women enrolled in this pilot showed 87% of women approached agreed to be part of the trial and there was 94.2% retention. All women received the dresssings they were randomized to, and inter-rater reliability for the outcome SSI was 0.87 (citation masked for blinded peer review).

### 3.1. Participants and Setting

This study took place in one Australian hospital. As this was a pilot study, the target sample size was set at 80–100 [20]. Inclusion criteria were: (i) women booked for elective CS surgery; (ii) recorded pre-pregnancy BMI of ≥30 and (iii) able to provide written informed consent. Exclusion criteria were: (i) women whose condition changes to warrant an urgent or emergency CS; (ii) previous participation in this trial; (iii) existing infection after admission to hospital and prior to CS; and (iv) unable to speak or understand English with no interpreter present.

### 3.2. Outcomes

The primary outcome for this study was surgical site infection (SSI), as defined by the Centers for Disease Control and Prevention [3]. Secondary outcomes included: (1) type of SSI–superficial incision, deep incision or organ/body space using the CDC criteria; (2) wound complications (*i.e.*, dehiscence, haematoma, bleeding, seroma, blisters); (3) hospital length of stay (HLOS); and (4) hospital readmissions (within 28 days). All outcomes except HLOS and readmission were assessed daily while the women were in hospital and weekly for 4 weeks after hospital discharge. No changes in the proposed trial outcomes occurred during the study.

### 3.3. Intervention and Control

At the completion of skin closure, those randomly allocated to the NPWT had, a PICO™ (Smith and Nephew, Hull, UK) applied by the obstetrician under sterile conditions. Women in the control arm had, Comfeel Plus^®^ (Coloplast, City, Denmark) dressing applied per manufacturer’s recommendations after skin closure. In both groups, the dressing remained in place until day 4, unless it became soiled or dislodged, in which case a new dressing of the same type was applied. To ensure consistency, obstetricians, nurses and midwives received trial-specific education (Negative Pressure Wound Therapy (NPWT) and Comfeel Plus standard dressing). The research assistant (RA) was available to clinical staff via telephone and in person to provide ongoing training and support about correct use of the dressings as well as monitor dressing changes and complete documentation daily to assess protocol compliance and outcomes.

### 3.4. Procedure

Potential participants were screened between the 32nd and 38th weeks of gestation by either the attending doctor or midwife in the antenatal clinic. An RA who was a Registered Nurse recruited participants during their 36th week outpatient visit, providing potential participants with an information summary of the research. If women agreed to participate, they signed a consent form. On the day of the elective CS, the RA confirmed ongoing consent from the women. Randomization was via a computer-generated 1:1 ratio, and had blocks of randomly varying sizes. Randomization occurred by the RA in the operating room. A centralized web-based randomization service was accessed which ensured allocation concealment.

The RA collected all outcome data daily while the women were in hospital. Following hospital discharge women were contacted weekly until the study end-point, at 28 days. Field notes were recorded that provided narrative information regarding the conduct of the trial and the care women received. A separate person, experienced in assessing for SSI, assessed the outcome SSI and was blinded to group allocation. Assessment of the data for SSI occurred at two intervals during the course of the study, firstly data on 35 women was assessed prior to preliminary analsysis (9 months into the trial) and the remaining 52 women’s data was examined on completion of the study. All women had completed 28 days of data collection at time of outcome assessment.

### 3.5. Data Analysis

Descriptive and infererntial statistics were used to analyse the data. Continous variables were summarized using mean and standard deviation (SD) or median and inter quartile range (IQR) based on normality assumptions. Normal continous varibles were compared between the intervention and control groups, using independent t-test while those that were not normal were analysed using Mann Whitney U test. Categorical variables were described using frequency and percentages. Testing of hypotheses of categorical varibles were evaluated using Chi-square test or Fisher’s exact test as appropriate.

Primary and secondary outcome variables were compared by computing the risk in each group and risk ratio (RR) and 95% confidence interval (CI). We did not expect statistical signficance between the groups for the oucome measures but point estimates (RR) were expected to show the direction and approximate magnitude of effect, if the study were to have been sufficienty powered. With the intention of conducting a larger trial, we used these data for a power calculation. Most data analyses were carried out using SPSS version 21 [21], MedCalc [22] was used for risk computations and confidence intervals, and PASS version 12 [23] was used for sample size calcuations.

## 4. Results

**Figure 1:**
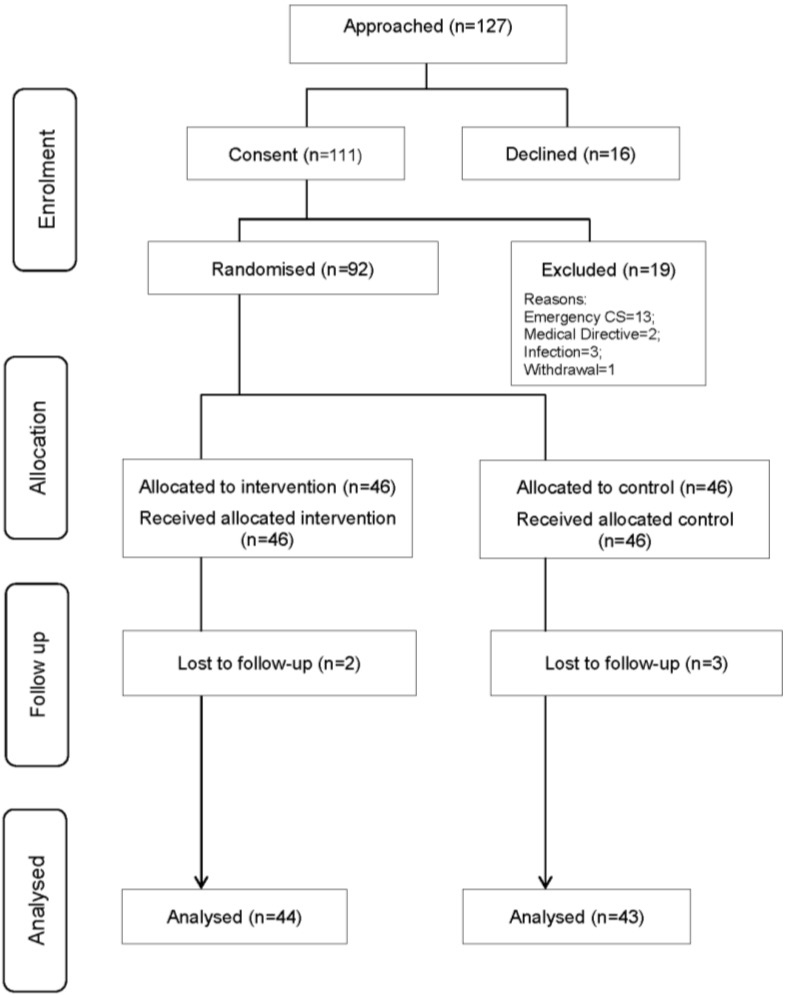
Participant Flow Diagram.

Recruitment occurred from July 2012 to April 2014. As identified in the flow diagram (Figure 1), a total of 111 women were recruited but 19 (17%) were subsequently excluded prior to randomisation. There was incomplete outcome data on 5 (5%) women, therefore the final analysis included 87 women. Four of the five women dropped out before the final data collection point and the fifth was transferred inter-hospital and had no outcome data. All women in the intervention and all women in the control group received the dressing to which they were randomized. One (2.2%) woman in the intervention group had a subsequent dressing change that resulted in a standard (rather than NPWT) dressing being used for the replacement (contamination). In successive dressing changes, none of the control women received the intervention (NPWT) dressing. Women were analysed according to their randomized dressing irrespective of whether they received a different dressing to the group to which they were allocated during the study period.

Table 1 displays the characteristics of the sample. While the two groups were similar, the length of surgery was longer in the control group and this group also had more smokers. The number of women requiring a dressing change was significantly different, with the 5/43 (11.6%) of the control group and 16/44 (36.3%) of the intervention group having at least one dressing change (*p* = 0.006).

**Table 1:** Characteristics of the Sample (*n =* 87).

Characteristic	Intervention Group *n =* 44	Control Group *n =* 43	*p*-Value
	Median (IQR)	Median (IQR)	
Age	30.6 (5.5)	30.7 (5.0)	0.925
Body Mass Index	35.7 (4.5)	36.8 (5.8)	0.538
Length of surgery (minutes)	45.0 (16.0)	53.0 (16.0)	0.002
	Frequency (%)	Frequency (%)	
^a^ Co-morbidities (yes/no)	30 (68.1)	30 (69.7)	−0.145 z score
Number of co-morbidities			
0	14 (31.8)	13 (30.2)	
1	18 (40.9)	21 (48.8)	
2	10 (22.7)	5 (11.6)	
3	1 (2.3)	3 (7.0)	
4	1 (2.3)	1 (2.3)	
Previous CS (yes/no)	37 (84.0)	40 (93.0)	−0.188 z score
Number of previous CS			
0	7 (15.9)	3 (7.0)	
1	24 (54.5)	28 (65.1)	
2	7 (15.9)	11 (25.6)	
3	5 (11.4)	0 (0.0)	
4	1 (2.3)	1 (2.3)	
Smoker	3 (6.8)	10 (23.3)	0.032
Diabetic (any type)	13 (29.5)	12 (27.9)	0.290

^a^ Comorbidities included: Anaemia, Diabetes Mellitus, Gestational Diabetes, Hypercholesterol, Hypertension, Immuno-compromised, Nutritional deficiency, Thromboembolytic disease, Smoking, Other.

Table 2 shows the comparison of primary and secondary outcomes. In total, 27.9% of the control group and 22.7% of the intervention group had a SSI, but this difference did not reach statistical significance, due to smaller sample size. However the RR of 0.81 (95% CI 0.39; 1.68) shows the risk of SSI was almost 20% lower in the NPWT group (10/44) compared to the control group (12/43) which may be clinically important, although not statistically significant with this sample size. As identified in Table 2, there were no statistically significant differences in the other outcomes, although there was a trend towards reduced bruising but increased blistering in NPWT. No women in either group experienced a seroma or dehiscence.

**Figure 2:**
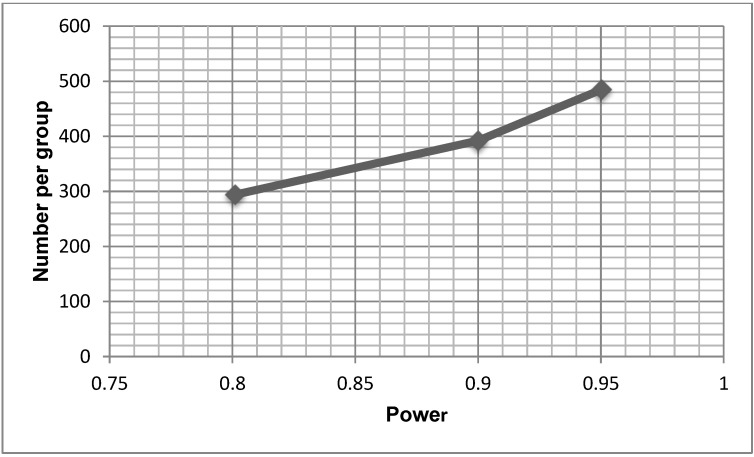
Power Curve.

Figure 2 provides a power curve based on the SSI data, demonstrating the various sample sizes required for trials powered at 80%, 90% and 95%. A sample size of 392 per group would be required to find a statistically significant difference in SSI between the two groups with 90% power.

**Table 2:** Relative Risk of Outcomes (*n =* 87).

Outcome	Intervention *n =* 44 Frequency (%)	Control *n =* 43 Frequency (%)	RR	95% CI	*p* Value
Surgical site infection	10 (22.7)	12 (27.9)	0.81	0.39–1.68	0.579
*Type of SSI*					^a^ 0.928
Superficial incision	5 (11.4)	7 (16.3)	0.70	0.24–2.03	0.509
Deep incision	4 (9.1)	4 (9.3)	0.98	0.26–3.66	0.972
Organ/space	1 (2.3)	1 (2.3)	0.98	0.06–15.13	0.987
Number of wound complications (excluding SSI)	6 (13.6)	6 (14.0)	0.98	0.34–2.79	0.966
Number of complications (including SSI)	14 (31.8)	17 (39.5)	0.80	0.46–1.42	0.454
*Type of wound complication*					^a^ 0.147
Bleeding	1 (2.3)	1 (2.3)	0.98	0.06–15.13	0.987
Bruising	1 (2.3)	4 (9.3)	0.24	0.03–2.10	0.199
^b^ Other	4 (9.1)	1 (2.3)	3.91	0.46–33.58	0.214
Hospital readmission	1 (2.3)	1 (2.3)	-	-	0.987
	Median (IQR)	Median (IQR)			
Hospital length of stay (days)	3.0 (1.0)	3.0 (1.0)	-	-	0.724

^a^ May be inaccurate due to number of cells with small expected values; ^b^ All other complications in the intervention group were blisters and the one other complication in the control group was erythema.

Effect size = 0.10, Baseline rate = 0.30, Alpha = 0.05, 2 sided.

## 5. Discussion

This pilot study was undertaken to assess the feasibility of a larger definitive trial comparing the use of NPWT to standard dressings including the likely sample size required to detect a significant difference in SSI. Our findings showed a trend towards fewer SSI in the NPWT group, although this difference was not statistically significant, likely due to the small sample. However, if this trend were to be supported in an adequately powered trial, it could have important implications for clinical practice. Preventing some SSIs has a number of benefits including improving women’s recovery from surgery, decreasing the need for treatment and hospital length of stay as well as saving valuable health care dollars. Argubly, despite NPWT being more expensive than conventional dressings, the benefits in preventing SSI may outweigh these dressing costs. It would be important for larger trials to incorporate some form of economic analysis, given the limited evidence in this area. This recommendation is also supported by a Cochrane Review of NPWT, which only identified one abstract of a study that considered costs but the full paper was not available [16]. There is however some costing research in other patient populations, with one study of patients with diabetic foot wounds finding that the average cost to achieve healing was less in the NPWT group (although this was a small study) [24]. Without good quality RCT evidence of effect or cost-benefit, it would be premature to recommend using NPWT for surgical wounds in obese women undergoing elective CS.

Despite our sample being randomized, there were group differences in both the smoking status of the women and the length of surgery, with women in the control group reporting smoking more and their surgery was longer. Both factors are recognised risk factors for SSI, thus it is always possible that these results explain the trend towards more SSI in the control group. It would be expected that randomising women in a trial with a larger sample size would see at least the difference in smoking status between the two groups disappear. In a larger sample, the length of surgery, a potential confounder, could be handled statistically, by entering it into the analysis as a covariate.The independent effect of NPWT could then be assessed while controlling for length of surgery. However, the clinical significance of an average of 10 min longer surgery is unknown.

The NPWT dressing was acceptable to both the women in the trial and the healthcare teams providing care. For example we were able to enrol 87% of the women approached to participate in the study and all women allocated to the NPWT received it (*i.e.*, clinicians did not prevent the NPWT dressing from being used in women recruited to the study). However, a general lack of familiarity with the NPWT dressing meant that ongoing education of both the medical and nursing staff was required. Additionally, at times when the research team was not available, some NPWT dressings were changed and in one instance, was replaced by the standard (control) dressing. In fact, 36% of the NPWT group had at least one dressing change, as compared to 12% in the control group. This unexpected finding requires further understanding as it has implications for future research, treatment costs and clinical practice. For example, this may indicate the need for additional staff training to ensure unnecessary dressing changes do not occur or it may indicate some other factor that can be addressed such as poor application technique.

An important consideration for RCTs is the extent to which the control and intervention groups receive similar care. In this hospital, women having elective CS followed a standardised care pathway during their admission, which should have standardised important aspects of care. However, the RA observed and documented subtle differences in certain aspects of surgical care such as antibiotic timing in theatre, surgeons’ preference for wound closure including suture materials, and type of standard dressing. Each of these issues could influence the findings of a definitive trial. Clinical practice guidelines and systematic reviews recommend pre-operative prophylactic antibiotics for clean contaminated wounds such as CS [25,26,27]. Including these recommendations during education sessions related to a larger trial may help to standardize practice. In terms of wound closure, the 2008 National Institute of Health and Clinical Excellence guidelines note there is no high quality evidence to recommend one practice over another [28], but a recent meta-analysis found closure with staples had a twofold higher risk of wound infection than closure with subcuticular sutures [29]. Thus, including the use of sutures rather than staples for wound closure in future trial protocols could reduce the potential impact of this potential confounder, although a small study of 63 women undergoing CS found surgeons preferred staples over sutures [30]. Finally, in terms of what dressings were used in the control group, a 2011 Cochrane review found no evidence to suggest one dressing type was better than others for the prevention of SSI [31]. It could be that there are differences that have yet to be demonstrated. In future trials, standardizing the dressing type in the control group may be prudent, but some variation in clinical practice does not mean that subsequent trials without standardisation cannot be completed in a rigorous manner. It does however suggest that future trials should be a pragmatic (versus explanatory) trial. Sackett suggests that pragmatic trials answer the question “Does this treatment improve patient-important outcomes when applied by typical clinicians to typical patients?” [32]. There are a number of features of pragmatic trials that make them particularly well suited for testing interventions such as wound dressings in the clinical environment. First, pragmatic trials focus on effectiveness in usual circumstances or practice. Second, the intervention is applied in a flexible way, as it would be in clinical practice. Finally, the findings of the research are generally directly relevant to patients, clinicians and decision makers. As part of the feasibility component of this trial, we generated information to estimate a range of possible sample sizes for the primary outcome of SSI, required for a larger definitive trial. Using this approach reflects best practice and has added to the methodological rigor of this pilot trial [33]. However, as there may be some uncertainties around sample size estimates obtained through pilot trials, it is also recommended to discuss estimates with clinicians to obtain additional information around clinically meaningful effect sizes [33]. Our results indicate that a definitive trial would require an overall sample size of 784 (*i.e.*, 392 per group in a two arm trial) to have 90% power to find a difference between groups if the primary outcome was the absence or presence of a SSI. Clearly, if SSI remains the primary outcome it will require a multi-site study.

In our pilot study we measured a number of other complications including bleeding, bruising, blister, seroma and dehiscence, but only noted whether they were present or absent and not the extent of each. Clinically, a small amount of bleeding, bruising or blistering would likely have little effect on the women or their ongoing care, but if more extensive, would likely require corrective action. Interestingly, there were no cases of either seroma or dehiscence reported but it is always possible this could occur in a larger sample. There was a trend towards more blistering in the NPWT group but none in the control group developing blisters. In one trial of 60 patients undergoing total knee arthroplasty, the rate of blisters in the NPWT group was so high (63%; RR 18.3 95% CI 4.3–77.6), the trial was stopped [34]. A recent review suggests skin blisters are common in orthopaedic surgery when adhesive dressings are used because of the swelling/oedema that occurs [19]. Clearly, blistering is an important safety consideration for both future trials and when the NPWT dressings are used in clinical practice.

An alternative option for selecting the primary outcome measure for the definitive trial is to develop a “composite” outcome such as “any wound complication” used in some previous research [18]. A composite measure involves aggregating the scores of several variables into an overall score [35]. The use of composite measures *versus* single outcome measures has been debated for some time [35,36,37]. Using a composite measure of “any wound complication” as the primary outcome in a definitive trial would likely result in a smaller sample size being required to demonstrate statistical significance. Nonetheless, there are also a number of limitations to such an approach. For example, grouping more serious complications like SSI and wound dehiscence with minor blistering or bleeding could make interpretation of the research findings including their clinical relevance difficult.

Other considerations for the larger definitive trial include standardizing training across sites especially proper application of the NPWT dressing, a clear monitoring plan to ensure the trial is proceeding as planned and additional data collection about site specific processes. Given the challenges associated with the real-world clinical settings, and the large number of health care providers involved in the clinical management of this population, using a pragmatic approach to trial design is appropriate.

## 6. Conclusions

This pilot study of 87 women showed that a larger definitive trial is feasible. Almost 90% of women approached agreed to be in the trial and 95% completed it. A sample size of 784 women would be required to detect a 20% difference in SSI at 90% power. A pragmatic trial, and associated process evaluation may be an appropriate approach if a definitive trial is undertaken in the future.
